# Reprogramming of sentinel lymph node microenvironment during tumor metastasis

**DOI:** 10.1186/s12929-022-00868-1

**Published:** 2022-10-20

**Authors:** Yen-Liang Li, Wen-Chun Hung

**Affiliations:** 1 National Institute of Cancer Research, National Health Research Institutes, Tainan, 704 Taiwan; 2 School of Pharmacy, College of Pharmacy, Kaohsiung Medical University, Kaohsiung, 807 Taiwan

**Keywords:** Lymph node, Immune, Metastasis, Microenvironment

## Abstract

Metastasis is a major cause of death in patients with cancer. The two main routes for cancer cell dissemination are the blood and lymphatic systems. The underlying mechanism of hematogenous metastasis has been well characterized in the past few decades. However, our understanding of the molecular basis of lymphatic metastasis remains at a premature stage. Conceptually, cancer cells invade into lymphatic capillary, passively move to collecting lymphatic vessels, migrate into sentinel lymph node (SLN;, the first lymph node to which cancer cells spread from the primary tumor), and enter the blood circulatory system via the subclavian vein. Before arriving, cancer cells release specific soluble factors to modulate the microenvironment in SLN to establish a beachhead for successful colonization. After colonization, cancer cells inhibit anti-tumor immunity by inducing the recruitment of regulatory T cell and myeloid-derived suppressor cells, suppressing the function of dendritic cell and CD8^+^ T cell, and promoting the release of immunosuppressive cytokines. The development of novel strategies to reverse cancer cell-triggered SLN remodeling may re-activate immunity to reduce beachhead buildup and distant metastasis. In addition to being a microanatomic location for metastasis, the SLN is also an important site for immune modulation. Nanotechnology-based approaches to deliver lymph node-tropic antibodies or drug-conjugated nanoparticles to kill cancer cells on site are a new direction for cancer treatment. Conversely, the induction of stronger immunity by promoting antigen presentation in lymph nodes provides an alternate way to enhance the efficacy of immune checkpoint therapy and cancer vaccine. In this review article, we summarize recent findings on the reprogramming of SLN during lymphatic invasion and discuss the possibility of inhibiting tumor metastasis and eliciting anti-tumor immunity by targeting SLN.

## Introduction

Cancer cells are highly proliferating cells that develop in heterogeneous environments. When tumors grow to a certain size, cancer cells face with oxygen/nutrient supply and metabolic waste accumulation. Escape from primary tumors (known as metastasis) is a selection pressure or an advantage for cancer cells to counteract the harmful microenvironment. Metastasis is a biological process that guarantees the sustained growth of cancer cells. It remains largely incurable and is responsible for up to 90% of cancer-associated mortality. The invasion-metastasis cascade of cancer cells is orchestrated by the following events: (1) invasion into the surrounding tissues containing abundant stromal cells and dense extracellular matrix (ECM); (2) intravasation into the circulation systems including the blood or lymphatic system; (3) survival in the circulation and travel to distant organs; (4) extravasation from blood vessels for colonization; (5) establishment of micrometastases to build a beachhead at the colonized sites; and (6) expansion in the new microenvironment to generate a secondary tumor.

## Metastatic spread via blood and lymphatic vessels

Cancer cells are mainly disseminated via the blood and lymphatic systems. The lymphatic system not only serves as a circulation system for the collection of interstitial fluid into the bloodstream [1], but also as an immune defense barrier to ensure the cleanliness of lymph fluid returning to the circulation. Terminal lymphatic vessels are thin-walled capillaries without pericyte coverage that are easier for cancer cells to penetrate. It has been proposed that approximately 95% of peritumoral vessels invaded by cancer cells are lymphatics [2, 3]. The lymphatic vasculature comprises lymphatic endothelial cells (LECs) that express molecular markers including lymphatic vessel endothelial receptor 1 (LYVE-1), prospero homeobox protein 1 (PROX1), podoplanin (PDPN), vascular endothelial growth factor receptor-3 (VEGFR-3), neuropilin-2 (NRP-2), and C–C motif chemokine ligand 21 (CCL21). Vascular endothelial growth factor-A (VEGF-A), -C, and -D released by cancer cells stimulate the growth of peripheral tumor lymphatic vessels and promote the invasion of cancer cells into nearby lymph nodes (known as sentinel lymph node, SLN) to enhance tumor metastasis [4–7]. Accumulating evidence suggest that VEGFR-3-mediated activation of LECs is a crucial step in the induction of lymphatic metastasis [8]. However, other factors, such as lymph flow rate, surface receptors expressed on tumor cells, and chemokines released from LECs also affect the entry of cancer cells to lymphatic vessels [9–11]. It is possible that these factors work together to promote lymphatic metastasis.

### The importance of lymph node invasion in tumor metastasis-a question still under debate

Animal studies have strongly supported the role of lymphangiogenesis and lymph node invasion in tumor metastasis. The orthotopic transplantation of VEGF-C-overexpressing breast cancer cells onto nude mice increased intratumoral lymphangiogenesis and significantly promoted tumor metastasis to the regional lymph nodes and lungs [12, 13]. Similarly, VEGF-A has been observed to trigger SLN lymphangiogenesis and lymphatic metastasis to enhance tumor spread [14, 15]. The ectopic expression of other lymphangiogenic factors, such as VEGF-D, platelet-derived growth factor-BB, and fibroblast growth factor-2, also induced metastasis in different cancer models [16–18]. In clinical setting, micrometastasis in SLN is a crucial factor associated with reduced distant metastasis-free survival and overall survival in cutaneous melanoma [19]. Leiter et al. also showed that dissection of SLN in primary melanoma decreased distant metastasis [20]. Scoring of immune and stromal features of SLN predicted distant metastasis in breast cancer patients [21]. In addition, lymph node metastasis increased the incidence of distant metastasis (hazard ratio = 3.495) in thyroid cancer [22]. By studying somatic variants in specific DNA regions to address the origins of lymphatic and distant metastasis, Naxerova et al. demonstrated that 36% of distant metastasis arose from lymph nodes in colorectal cancer [23]. Similarly, phylogenetic investigation also showed that 25% of metastatic tumors at distant sites were derived from lymph node metastasis [24]. A very recent study clearly demonstrated that colonization of lymph nodes by cancer cells elicited a chronic interferon signaling and triggered antigen-specific immune tolerance to promote distant metastasis [25]. However, several clinical intervention studies showed that prophylactic LN removal does not improve overall survival in melanoma [26], thyroid cancer [27], and breast cancer [28]. A long-term follow-up of a randomized trial demonstrated that localized lymph node metastasis, distant metastasis and patient’s survival were not strongly corelated [29]. In addition, lymph node-negative colorectal cancer patients had higher incidence of lung metastasis [30]. In contrast, a prospective multicenter study of early stage endometrial cancer suggested that SLN biopsy provides important information to for tailoring adjuvant therapy [31]. SLN biopsy continues to be a critical procedure in the clinical management of patients with salivary gland tumors [32]. It should be noted that clinical outcome of lymph node-positive patients is often worse than that of lymph node-negative patients in some types of human cancer. For instance, in clinical stage IIB and IIC melanoma patients, SLN status is the most important prognostic factor and positive SLN involvement is strongly correlated reduced distant recurrence-free survival and disease-specific survival [33]. Similar finding was reported in a retrospective cohort of 2086 melanoma patients [34]. In addition, a national cohort study in which 8562 patients were included also concluded that SLN status is a critical prognostic factor in stage IIB/C melanoma patients [35]. In colon cancer, the average survival of lymph node-positive patients was shorter than that of lymph node-negative patients (66 vs. 89 months) [36]. In addition, the number of positive lymph nodes affected the response of patient to chemotherapy. The average survivals of patients with 1-, 2-, and 3-positive lymph nodes who received chemotherapy were 108, 83 and 54 months respectively. In pancreatic cancer, lymph node-negative patients have a longer median overall survival than lymph node-positive patients (25 vs. 16 months) [37]. In esophageal cancer, lymph node-positive patients with tumors localized at middle and lower regions had a worse prognosis than the lymph node-negative patient [38]. Emerging evidence suggests that tumor metastasis may occur at a very early stage of tumorigenesis. Therefore, resection of the primary tumor and SLN may not significantly improve patient survival if cancer cells have already been seeded on distant organs via the hematological and lymphatic systems. As aforementioned in the studies of evolutional relation between primary tumor, lymph node metastasis and distant metastasis [23, 24], around 30–40% of distant metastasis arose from lymph node metastasis. Many cancers may metastasize to distant organs via blood and lymphatic systems simultaneously and the contribution of lymph node metastasis to distant dissemination could be cancer type-dependent. Moreover, other important factors including the number of lymph nodes evaluated, the number of positive lymph nodes, the features of lymph node microenvironment and the depth of lymph node involvement all affect the results of pathological evaluation and the conclusions of clinical association. For example, a systematic review of a total 61,371 colon cancer patients showed that the number of surgically dissected lymph nodes evaluated was positively correlated with the survival of stage II and III patients [39]. Enhancement of angiogenesis and lymphangiogenesis in the SLN was found to be linked with distant metastasis and survival of melanoma patients [40]. In addition, the number of B cells in the SLN, regardless of the status of cancer cell invasion, also predicted disease-free survival in patients with breast cancer [41]. Therefore, the importance of lymph node invasion in the induction of distant metastasis warrants continuous study.

### Lymph node structure

The lymphatic system comprises a large network of lymph and lymphatic capillaries, collecting lymphatic vessels, lymph nodes and lymphoid organs. Lymph, the fluid that drains from cells and tissues, contains small molecules (minerals and amino acids), large molecules (proteins and lipids), and cells (damaged cells or immune cells). It flows from the lymphatic capillary, the terminal vessel of the lymphatic network, to a large collecting lymphatic vessel that further connects to the lymph node, a kidney-shaped organ of the lymphatic system. Lymph nodes are classified as "secondary" lymphoid organ, while the primary lymphoid organs comprise the thymus, tonsils, spleen, and bone marrow. Approximately thousands of lymph node are linked throughout the body by lymphatic vessels [42] and are particularly distributed in the chest, neck, pelvis, axilla, and inguinal region, and in association with the blood vessels of the intestines [43]. The anatomical structure of the lymph node is divided into several compartments. The outer portion of the lymph node consists of the cortex, containing the B-cell follicle, and the paracortex, containing the T-cell zone. The inner portion of the node is the medulla which contains blood vessels, sinuses, and medullary cord. Antibody-producing plasma cells, macrophages, and B cells are the major cell types in the medullary cord. A specialized structure, high endothelial venules (HEVs), found in the paracortex are the main routes for lymphocytes to enter the lymph node.

The SLN is defined as the first lymph node with direct lymphatic flow from the primary tumor and is the beachhead for the earliest stage of lymphatic metastasis. Clinically, the presence of tumor cells in the SLN is a prognostic factor associated with cancer progression and poor patient outcome [44–46]. The biological features of SLN include the enhancement of lymphangiogenesis, increase in lymph flow [47], structural remodeling of HEVs [4, 48], enhanced recruitment of myeloid cells, and reduction of effector lymphocytes [49], all of which contribute to the establishment of a pre-metastatic microenvironment for the entry and survival of cancer cells.

### Reprogramming in immune cells in the SLN

**Fig. 1:**
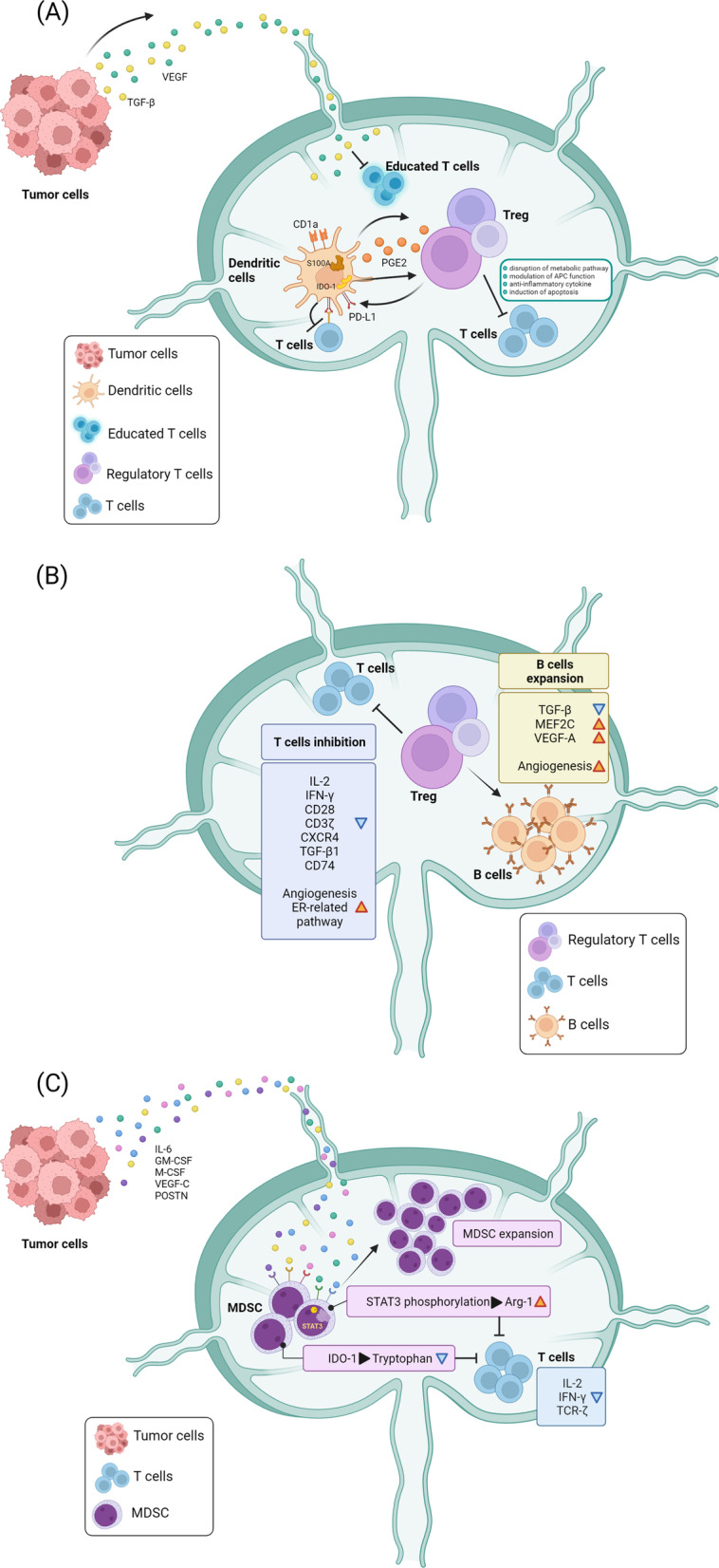
Immune microenvironment modulation in the SLN during cancer progression. **A** Tumor cell-derived soluble factors including TGF-β and VEGF impair T cell and lymphocyte activation by DCs. Expression of COX2 and IDO-1 in DCs also contribute to the expansion of Treg cells which directly diminish T cell activity. Treg cells stimulate a feedback loop to enhance PD-L1 expression on DCs, leading to immune suppression of T cells via the receptor PD-1. S100 and CD1a elevated in the DCs in the SLN also modulate antigen presentation of DCs. **B** After expansion, Treg cells enter the SLN via HEV and lymphatic vessels. Treg cells inhibit T cell activity, decrease pivotal molecules such as IL-2, IFN-γ, CD28, CD3-ζ chain, CXCR4, TGF-β1 and CD74 to suppress the proliferation, activation and differentiation of T cells. Treg cells also induce B cells expansion and enhance MEF2C expression to alter the proliferation and survival of B cells. In addition, Treg cells promote the estrogen receptor-related pathways in T cells to facilitate angiogenesis and lymphangiogenesis. **(C)** MDSCs are recruited to the SLN and are activated by external signals such as IL-6, GM-CSF, M-CSF, VEGF-C and POSTN released from distant tumor cells. STAT3 phosphorylation in MDSCs stimulate the production of Arg-1 to compete the essential amino acid with T cells and to suppress T cell function. In addition, IDO-1-expressing MDSCs catalyze the metabolism of tryptophan to kynurenine, a metabolite which induces T cell apoptosis

The immune microenvironment in the lymph node is orchestrated by immune cells including macrophages, dendritic cells (DCs), T cells, B cells, and non-immune cells, such as fibroblastic reticular cells (FRCs), blood endothelial cells (BECs), and LECs. One of the key regulators of the host immune system to attack cancer cells are DCs, highly specialized antigen-presenting cells, that play a crucial role in the initiation of cellular immunity (Fig. 1A). Previous studies have demonstrated that the anti-cancer activity of T cells is dramatically attenuated in the absence of DC [50–53]. In the SLN, DC-induced T-cell activation is significantly impaired by cancer cells via direct cell–cell contact or cancer cell-secreted factors, such as transforming growth factor-β (TGF-β) and VEGF. Munn and Sharma et al. showed that a small population of indoleamine 2,3-dioxygenase 1 (IDO1)-expressing plasmacytoid DCs in the SLN was capable of inducing regulatory T-cell (Treg) generation and T-cell anergy, which was linked with decreased T-cell response to tumor antigens [54, 55]. Sakakura et al. also demonstrated that the increase in S100^+^ and CD1a^+^ DCs in the SLN of patients with oral cancer suppresses immune response [56]. In addition, DCs have been reported to produce cyclooxygenase-2-derived prostaglandin E2 to promote the accumulation of Tregs in the SLN [46].

Lymphocytes are recruited into the lymph node mainly via the HEVs and lymphatic vessels. A previous study demonstrated a marked decrease in the number of CD4^+^ T helper (Th) cells in the advanced clinical stage of melanoma [57]. A reduction in lymphocyte infiltration in head and neck cancers has also been reported [56]. In addition, lymphocytes in the SLN of oral cancer were found to express distinct immune molecules, suggesting phenotypic alterations in these cells [58]. In parallel, another important sub-population is the immunosuppressive CD4^+^CD25^hi^ forkhead box P3 (FOXP3^+^) Tregs (Fig. 1B). These predominant cells are significantly elevated in tumor cell-positive lymph node in patients with different cancers [59, 60]. Tregs inhibit the proliferation of CD8^+^ T cells and weaken the ability of lymphocytes to produce interleukin (IL)-2 and interferon (IFN)-γ. The heterogeneity of T cells in primary tumor and SLN is very significant. Only a very small number of expanded T cells have been found in the SLN [48, 49]. Additionally, CD4^+^ T cells were dramatically decreased in the SLN in breast cancer [61, 62]. A similar finding has also been reported in oral cancer [58]. The accumulation of B cells in the SLN has been found to be associated with lymphangiogenesis and increased lymph flow, thereby effectively promoting the dissemination of lymphomas and solid tumors [63]. The increase in B cells in patients with cancer indicated the activation of clonal expansion, probably triggered by the recognition of tumor antigens, and suggested the enhancement of the apoptosis-inducing ability of B cells [64, 65].

Recently, we performed single-cell RNA sequencing to explore alterations in gene expressions in different cell populations in the SLN and identified the molecular pathways altered in T cells in the SLN [66]. Our results showed that angiogenesis-related gene sets were significantly upregulated in Cd4^+^, and Cd8^+^ T-cells and Tregs. Consistent with our findings, a recent study demonstrated an increase in the proportion of T cells, B cells, DCs and natural killer (NK) cells in metastatic lymph nodes [67]. Interestingly, the accumulation of N2 type neutrophil (Cd54^low^) was significantly enhanced. Moreover, the estrogen response gene signature, which is involved in enhancing breast cancer progression, was elevated in Cd4^+^ T cells in the SLN. Estrogen receptor 1 (Esr1), a transcription factor that binds directly to the gene promoter of retinoic acid-receptor-related orphan nuclear receptor γ, may suppress the differentiation and function of Th17 cells [68]. Moreover, Esr1 may inhibit follicular helper T-cell activation to prevent autoimmunity [69]. Our finding that Esr1 signaling is activated in Cd4^+^ T cells suggests a suppressed immunity in the SLN. Clusters of differentiated genes by gene set enrichment analysis using molecular signatures database revealed that the gene sets related to Foxp3-mediated Treg transcriptional regulation in the C7 immunological signature were altered, suggesting the enhancement of Treg activation and generation of an immune suppressive environment in the SLN. The expression of genes related to proliferation (Cxcr4), polarization (Cxcr4, Tgfb1, and Cd4), and differentiation (Cd74 and Cd4) in Cd4^+^ T cells in the SLN was reduced. The differentiation marker Cd74 was also downregulated in Cd8^+^ T cells in the SLN. Our results also demonstrated enhancement of the angiogenesis pathway in B cells, consistent with previous findings that tumor-associated B cells contribute to tumor progression by stimulating angiogenesis [70]. An increase in myocyte-specific enhancer factor 2C (Mef2c) in B cells was found in the SLN. Mef2c is a key transcription factor that increases B-cell proliferation and survival [71, 72]. In addition, this molecule protects B cell lymphopoiesis under stress conditions by regulating B-cell specific gene expression [73]. Finally, TGF-β, a critical cytokine in controlling the development of B cells from pre-B cells to immunoglobulin-secreting plasma cells [74], was downregulated in the SLN, indicating inhibition of functional differentiation.

Myeloid-derived suppressor cells (MDSCs) also participate in the enhancement of tumor growth and metastasis by accumulating in the SLN to suppress immune reactivity (Fig. 1C). Cancer cells secret IL-6, VEGF, macrophage colony-stimulating factor (M-CSF), and granulocyte-M-CSF (GM-CSF) to promote MDSC expansion and enhance their recruitment to SLN [75, 76]. MDSCs influence immune responses by (1) inducing the development and expansion of Tregs [77–79]; (2) depriving amino acids that are essential for T-cell growth and differentiation [80, 81]; (3) releasing oxidizing molecules, including hydrogen peroxide (H_2_O_2_) and peroxynitrite (ONOO^–^) to increase immune cell apoptosis [82, 83], and (4) interfering with T-cell migration [84, 85]. By increasing IDO1 expression, MDSCs decrease the immune response of T cells and trigger T-cell apoptosis via kynurenine production [86, 87]. These studies highlight the role of MDSCs in immune suppression and suggest the possibility of targeting MDSCs to overcome the immune escape. Macrophages are another myeloid lineage cell considered to be involved in lymph node metastasis. Broadly speaking, there are two main groups of macrophage designated as M1 and M2. M1 macrophages release inflammatory cytokines, whereas M2 macrophages exhibit anti-inflammatory activity. Tumor-associated macrophages (TAM) acquire an M2 phenotype that contributes to tumor growth and progression. It had been demonstrated that TAM level is significantly associated with pathologically positive-lymph node and is linked with enhanced lymphangiogenesis in the SLN [88]. In various types of cancer, the reduced expression of a unique type of CD169^+^ macrophages in the SLN has been correlated with poor clinical outcome [89–91]. Collectively, existing data suggest that the immune microenvironment in the SLN is conditioned prior to cancer cell arrival, and re-activation of anti-cancer immunity in the SLN may prevent tumor metastasis.

### Reprogramming in BECs/HEVs in the SLN

**Fig. 2:**
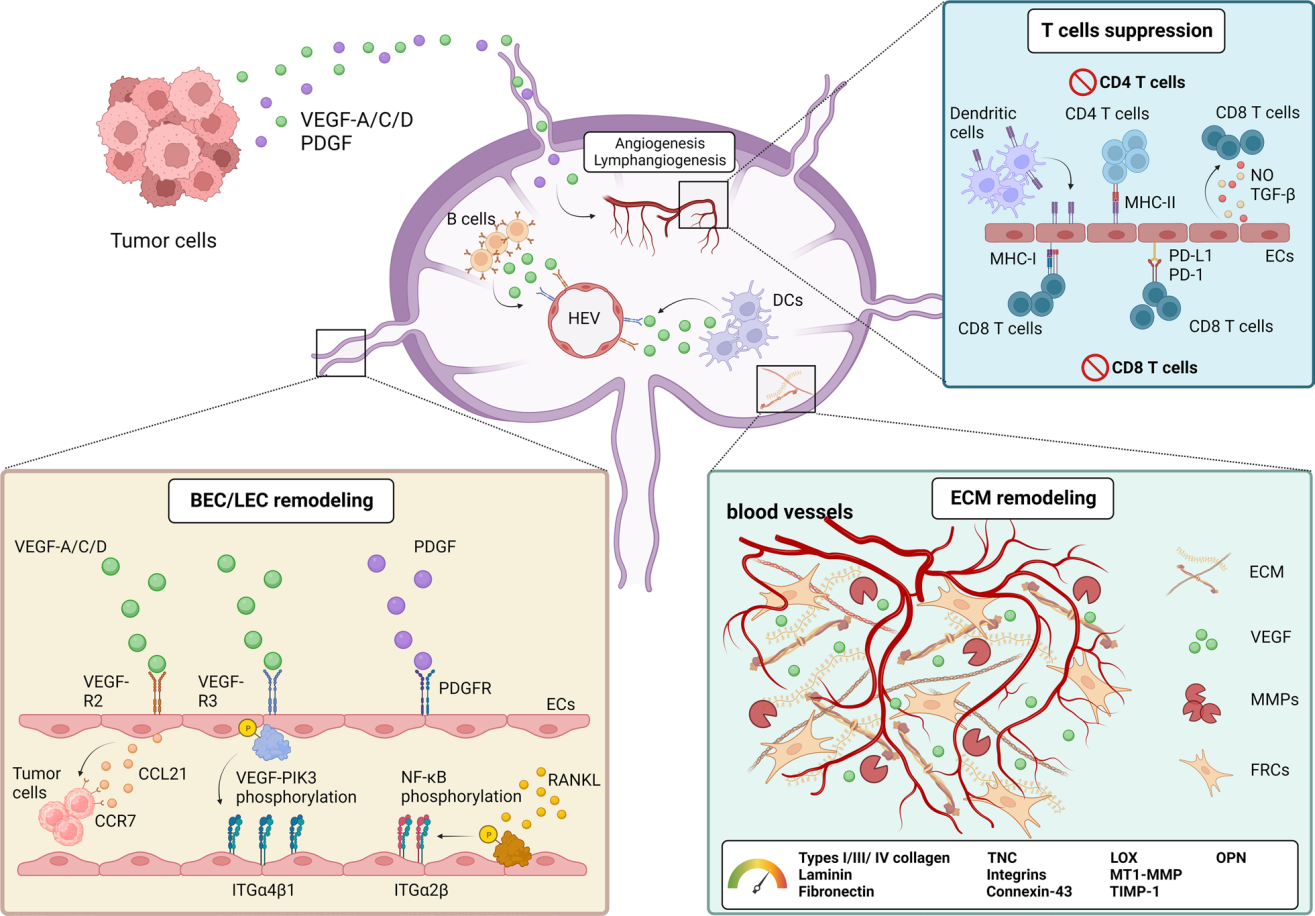
Endothelial cell reprograming in the SLN during cancer progression. Tumor cell-derived VEGF and PDGF directly activate their specific receptors on LECs. Signals induce ITGα4β1 expression via the VEGFR-3/PI3K axis. VEGFR-3 activation also contributes to CCL21 production by LEC, further attracting CCR7-expressing tumor cells homing to the SLN. PDGF from tumor cells stimulates the proliferation of LEC, providing more opportunities for tumor cells to establish the pre-metastatic niche. LEC activation by RANKL in the SLN enhances ECM remodeling to trigger the sprouting of LEC and BEC to enhance tumor metastasis to secondary lymphoid organ. B cells and DCs also secrete VEGF-A to remodel the BECs in HEV, leading to the decrease in vessel wall and the increase in HEV diameter which facilitate tumor cell metastasis and provide nutrient for sustaining tumor cell growth in the SLN. MHC II complexes acquired from DC present on cell surface of LEC induces apoptosis of CD4^+^ T cells. CD8^+^ T cell proliferation is also suppressed by the PD-L1 molecule expressed on LEC. Moreover, LEC can generate soluble factors such as nitric oxide and TGF-β to inhibit CD8^+^ T cell activation

The vascular endothelium plays a central role in the regulation of oxygen/metabolite exchange and recruitment of immune cells to lymphoid tissues. The vascular system of lymph node in mammals consists of arteries, capillaries, post-capillary venules and vein [92]. Recently, two transcriptome analyses of different murine organs revealed molecular markers of BECs in lymph nodes according to their location [93, 94]. HEVs are specialized post-capillary vessels with high expression of peripheral node addressin (PNAd), Ccl21 and Cd105 [95, 96]. These molecules are important for the selective recruitment of lymphocytes to the lymph nodes. Loss of PNAd in metastatic lymph nodes has been shown to affect lymphocyte homing [4]. Lymphocyte recruitment into the SLN occurs mainly through HEVs and partially from the draining afferent lymphatic vessels. The remodeling of HEVs (Fig. 2) in the SLN decreases the recruitment of lymphocytes and promotes the establishment of a pre-metastatic niche [97]. Studies in human samples have found reduced vessel wall thickness and increased vessel diameter in HEVs in the SLN [4, 98, 99].

**Fig. 3:**
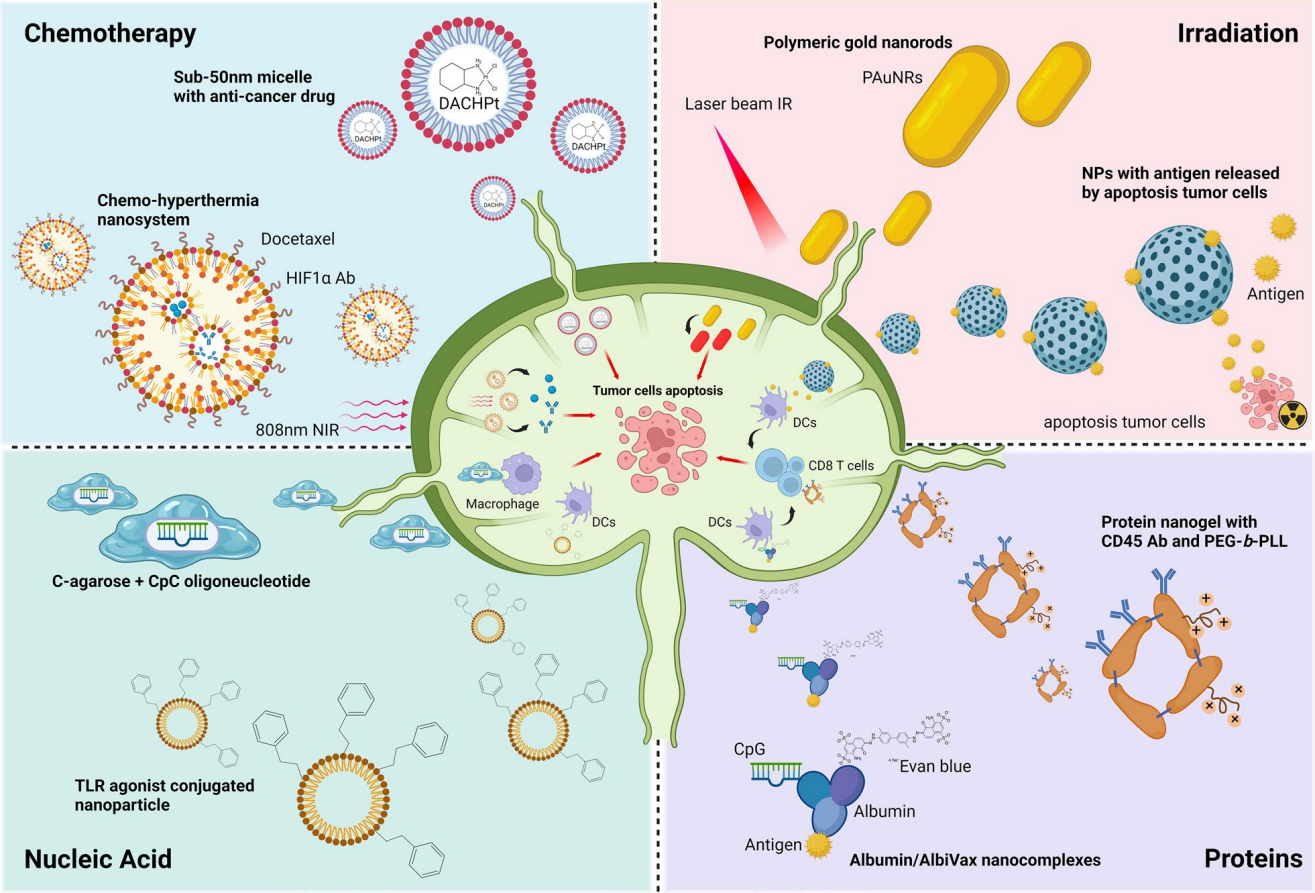
Novel LN-targeted therapeutic strategies. Nanoparticles have been developed to kill tumor cells or re-activate antitumor activity in the LN. For chemotherapy, 808 nm NIR-triggered nanosystem achieves synergistic chemo-hyperthermia effects to eliminate tumor cells in the metastatic lymph nodes. Nanocarriers can bring nucleic acid toward the LN. CpG oligodeoxynucleotides equipped with C-agarose display high affinity to the macrophages in the lymph node sinus and effectively trigger anti-tumor immune responses in the LN. TLR agonist-conjugated nanoparticles activate DCs in the LN and stimulate T cell activity. In radiotherapy, gold nanorods under short-term NIR laser irradiation may increase tumor cell apoptosis via a thermodynamic effect. Tumor antigens released from cancer cells after IR treatment can be utilized as cargos and carried by nanoparticles to the LN, thus enhancing antigen presentation by DCs to activate T cells. Nanogels carrying an IL-15 superagonist complex coated with CD45 antibody can bind to CD8^+^ T cells specifically and efficiently stimulate the proliferation of T cells by IL-15 stimulation. On the other hand, by conjugating albumin-binding vaccines with Evans blue, this nanocomplex provides another way for vaccine delivery and cancer immunotherapy

VEGF plays a pivotal role in stimulating BEC proliferation. This angiogenic factor not only acts as a survival factor for endothelial cells, but also stimulates them to degrade the ECM for sprouting and migration. Therefore, structural and functional alterations in the BECs of the SLN also accelerate lymphatic metastasis (Fig. 3).

### Reprogramming in LECs in the SLN

LECs are defined as a specialized population of endothelial cells that comprise lymphatic vessel in lymph node, which are faithfully characterized by PDPN and LYVE-1 and are localized to the subcapsular, cortical, and medullary sinuses [100–102]. Among the pro-lymphangiogenic factors, VEGF-C exhibits the most potent activity in inducing lymphangiogenesis, and VEGFR3 on LECs is the major receptor involved in tumor-associated lymphangiogenesis and lymphatic metastasis [102, 103]. Recently, single-cell transcriptomic analysis revealed different subtypes of murine LECs in skin-draining lymph nodes, suggesting the complexity of their function [104].

Currently, VEGF-A and VEGF-C are the most well-documented soluble factors correlated with the establishment of a pre-metastatic microenvironment in the SLN [5, 105]. The remodeling of LECs via the VEGF-C-PI3K axis is critical for tumor-associated lymphangiogenesis. This signaling pathway enhances integrin α4β1 expression on LECs to attract Vcam-1-expressing tumor cells [106]. Simultaneously, VEGF-C also enhanced the expression of Ccl21 in the lymphatic endothelium to promote the entry of Ccr7^+^ cancer cells into the SLN [107]. In contrast, lymphangiogenesis has been found to increase the lymph flow rate, which also accelerates lymph node metastasis [49, 108–110]. LECs in the SLN also expressed higher levels of receptor activator of nuclear factor kappa-Β (Rank), and stromal reticular cells activated LECs via RANK ligand (Rankl) to induce LEC remodeling [111].

The functional reprogramming of LECs also affects their immunomodulatory activities. Under physiological circumstance, LECs may present a variety of peripheral tissue antigen on major histocompatibility complex (MHC) class I molecules to induce immune tolerance and modulate Cd8^+^ T cell proliferation through programed death protein 1 (Pd-1)/programmed death-ligand 1 (Pd-l1) signaling [112]. In the SLN, by presenting soluble tumor-associated antigens from the lymph, LECs could trigger dysfunction in Cd8^+^ T cell and increase T-cell apoptosis by regulating the expression of Pd-1, Cd80 and cytotoxic T-lymphocyte associated protein 4 (Ctla4) [112]. In addition, LECs express MHC class I on the surface, leading to functional alteration of Cd8^+^ T cells [53, 113]. Moreover, LECs may acquire peptide/MHC II complexes from DC and present on their cell surface to trigger apoptosis of Cd4^+^ T cells [112]. Moreover, LECs can present tumor antigens and produce immunosuppressive molecules such as kynurenine, nitric oxide, and TGF-β to construct an immunosuppressive microenvironment in the SLN [53, 112–118].

### Reprogramming in stromal cells in the SLN

Lymph node stromal cells comprise distinct cell types: lymphoid tissue organizer (LTo), follicular DCs (FDCs), FRCs, marginal reticular cell (MRCs), integrin α7 pericytes (IAPs), BECs, and LECs. LTos recruit hematopoietic cells to lymph node. FDCs found in the cortex around the B cell zone of lymph node are important for supporting B cell survival. FRC produce various ECM proteins including fibrillary type I and III collagen, collagen type IV, laminin, fibronectin, tenascin-C, and integrins to form a reticular venue for immune cell movement and to strengthen the lymph node structure [119, 120]. FRCs play a crucial role in maintaining HEV integrity and immune cell survival. MRC constitutively produces chemokine CXCL13 to modulate the characteristic and functionality of LTo cells. IAP is a newly identified type of stromal cells located around blood vessels in the lymph node with uncharacterized biological function.

The reprogramming of stromal cells in the SLN has been previously reported in a B16.F10 melanoma mouse model [121]. This study demonstrated that genes participating in diverse biological processes, including growth, metabolism, mitochondrial function, cell motility, and cell–cell junction, were upregulated in FRC in the SLN of tumor-bearing mice when compared to naïve lymph node. Both gene set enrichment analysis and interpretative phenomenological analysis identified upregulated expression of genes encoding chemokines, cytokines and their downstream mediators in the SLN while several factors such as IL-19, IL-7, CCL4, and CCL21 were downregulated in comparison with those in naïve lymph node. Transcriptional profiling also showed that the expression of activation markers, including PDPN, fibronectin 1, Cd248, actin α2, S100A4, vimentin, myosin light chain, and collagens, was enhanced, indicating the activation of FRCs in the SLN.

Based on our single-cell RNA sequencing data, FRC in the SLN expressed a strong elevation of the oxidative phosphorylation (OXPHOS) pathway signature [55]. Twenty-seven genes of complex I, one of complex II, six of complex III, nine of complex IV, and four of complex V of the respiratory chain were differentially expressed, suggesting massive ATP consumption in the SLN. These data suggest that a metabolic switch in the SLN promotes lymphatic metastasis.

### The mediators for SLN reprogramming

#### Growth factors and chemokines

Mounting evidence suggests that distinct chemokine-receptor signaling pathways contribute to the trafficking of cancer cells to the lymph node (Table 1). Compared to naïve lymph nodes, the expression of GM-CSF, IFN-γ, IL-2, and IL-10 is elevated in the SLN in melanoma [122, 123]. In breast cancer, the expression of CD83, IL-12p40, IFN-γ, IL-10, and Foxp3 was evidently upregulated in the tumor-infiltrated SLN [124]. In non-small-cell lung cancer, tumor-derived TGF-β reduces the number of DCs in the SLN [53]. Hirakawa et al. reported that primary tumors overexpressed VEGF-A to induce lymphangiogenesis in the SLN before cancer cell arrival [5]. Chemokine receptors such as CXCR3 and CXCR4 have been shown to be upregulated in animal models of different types of cancer and have been strongly associated with SLN metastasis [100]. Das et al. found that CCL1 protein was detected in the lymph node and lymphatic sinuses, and CCR8, the cognate receptor for CCL1, was significantly upregulated on the cell surface of human melanoma cells, providing a molecular basis for how CCL1 promotes cancer cell invasion into the SLN [125]. Several paired ligand-receptor pathways, including CXCL12-CXCR4, CCL19-CCR7 and CCL21-CCR7, also effectively promote the lymph node invasion of cancer cells [126–128]. These studies imply that the cytokine/chemokine milieu plays a crucial role in establishing a pre-metastatic microenvironment in the SLN.

**Table 1:** Cytokines and chemokines modulation in SLN during cancer progression

Cancer type	Proteins and nucleic acids	Effect	Refs.
Cytokines			
	COX-2	Macrophages phenotype changes	
	GM-CSF	Proliferation and differentiation of DCS	[5]
Melanoma	IFN-γ	Production of cytotoxic cells	[122]
	IL-10	Dampens acquired Th1 and Th2 cell cytokine production	[123]
	VEGF-A	Lymphangiogenesis	
Breast cancer	Il-12p40		[124]
	IFN-γ		
NSCLC	TGF-β	Decreased DCS concentration	[53]
Chemokines			
Oral, breast,	CXCR3	Tumor-LEC chemotaxis	[100]
melanoma	CXCR4-CXCL12	Tumor-LEC chemotaxis	[126]
Melanoma	CCR8-CCL1	Tumor-LEC chemotaxis	[125]
Gastric, colorectal,	CCR7-CCL19/MIP-3β	Tumor-LEC chemotaxis	[127]
breast cancer	CCR7-CCL21/6Ckine	Tumor-LEC chemotaxis	[128]

### Extracellular vesicles

Extracellular vesicles (EVs) are double layer lipid-containing vesicles that are naturally released from most types of cells. EVs can be divided into exosomes, microvesicles and apoptotic bodies based on their size and synthesis route [129]. These vesicles play important roles in cell–cell communication; they deliver different bioactive molecules from secreting cells to recipient cells to modulate the behaviors of recipient cells under various physiological and pathological conditions. Recent studies have highlighted the mechanisms of exosome-mediated processes in the preparation of pre-metastatic niches for lymphatic metastasis [130–132]. Tumor-secreted EVs can rebuild the surrounding matrix and reprogram the microenvironment in the lymph node to establish a beachhead for cancer cell spreading (Table. 2) [133–135]. For example, laminin 332, a large ECM protein complex, was found to be increased in the exocrine bodies of tumor tissues of patients with oral cancer with positive lymph node metastasis. Depletion of this protein potently suppresses EV-mediated LEC migration and lymphangiogenesis in the SLN [136]. Interferon regulatory factor 2 detected in plasma EVs is taken up by F4/80^+^ macrophages to induce the release of VEGF-C to promote lymphangiogenesis and lymphatic network remodeling of the SLN in patients with colorectal cancer [137]. CD169^+^ macrophages captured microvesicles derived from B16-F10 melanoma cells to trigger microenvironment reprogramming after their entry into the SLN [138]. Enhanced IL-6 expression in macrophages induced by breast cancer-released EV suppresses immune response and promotes cancer metastasis in a xenograft mouse model [139]. In addition, EV containing PDPN promotes tube formation in cultured LECs [140]. Moreover, exosomes derived from cancer cells can transmit EGFR to endothelial cells and LECs to stimulate angiogenesis and lymphangiogenesis [141]. In addition to proteins, nucleic acids in EVs have been found to be involved in lymph node metastasis. In cervical squamous cell carcinoma, miR-221-3p expression correlates with LYVE-1 expression and lymph node metastasis [142]. Cancer cell-derived miR-containing EVs enhance the recruitment of MDSC and the activation of TAM to generate an immunosuppressive environment, resulting in increased cancer cell metastasis [132, 143]. Melanoma-released exosomes delivered miR-9 to endothelial cells to activate Janus kinase 2-signal transducer and activator of transcription 3 signaling in endothelial cells to trigger angiogenesis [144]. These results suggest that EVs are critical mediators for cancer cells to remodel the SLN microenvironment.

**Table 2:** SLN reprogramming with different cargo in EVs

Cancer type	Cargo in EVs	Effect	Refs.
OSCC	Lamin-332	Lymphangiogenesis	[136]
Colorectal cancer	IRF-2	Ingested by macrophage/induce VEGF-C expression	[137]
Breast cancer	Palmitoylated protein	NFκB activation via TLR-2 on macrophages	[139]
MDCK (normal kidney)	PDPN	Lymphangiogenesis	[140]
Melanoma, lung cancer,	EGFR	Angiogenesis	[141]
colorectal cancer			
Cervical cancer	miR221-3p	Induce LYVE-1 expression	[142]
Melanoma	PEDF	Recruitment of MDSCs/activation of TAM	[132, 143]
Melanoma	miR-9	Regulate angiogenesis via JAK-STAT	[144]

### Metabolites

The lymph node is a lipid-rich organ because the lymph draining into it contains many fatty acids [44, 145]. Lee et al. reported that cancer cells undergo a metabolic shift toward fatty acid oxidation (FAO) via selective activation of a transcriptional coactivator, yes-associated protein (YAP), to promote lymph node metastasis [145]. FAO also plays a crucial role in controlling lymphangiogenesis [146]. Furthermore, FAO affects multiple cell types in the SLN. For instance, a recent study revealed the importance of FAO upregulation through STAT3 activation in inhibiting CD8^+^ T-cell and in promoting obesity-associated breast tumorigenesis and metastasis [147]. The transcription factor Foxp3 increased the expression of acetyl-CoA synthetase and carnitine palmitoyltransferase 1A in Tregs, suggesting that Tregs prefer to use fatty acids as a major energy source; this process may contribute to functional reprogramming in the SLN [148]. Our study also demonstrated that bile acid metabolism and fatty acid metabolism are the hallmark pathways upregulated in the SLN. We found that overexpression of fumarylacetoacetate hydrolase (FAH) in FRCs induced by breast cancer cells significantly increased mitochondrial OXPHOS levels and ATP production. FAH catalyzes the hydrolysis of 4-fumarylacetoacetate to acetoacetate and fumarate, which can be metabolized in the tricarboxylic acid cycle or used for biosynthetic purposes. Co-culture of immune cells isolated from mice with FAH-overexpressing FRCs inhibited immune cell activation in vitro, suggesting that metabolic reprogramming in FRCs produces metabolites that generate a tumor-induced immunosuppressive niche in the SLN. The concept of “oncometabolites” has been well established by the finding that metabolites, such as fumarate, succinate, and 2-hydroxyglutarate, can induce epigenetic alteration, enhance cellular transformation and generate a favorable microenvironment for tumor progression [149].

### Extracellular matrix

The ECM encompasses many extracellular macromolecules, including collagen, proteins, and hydroxyapatite. It generates a three-dimensional network that serves as a structural and biochemical basis to support tissue integrity. Under physiological circumstances, FRC is the major source of ECM production in the lymph nodes. They produce distinct types of ECM to form specific conduits in the lymph nodes to transport bioactive molecules and to speed up the migration of immune cells within lymph node [119, 120, 150]. Recently, Wei et al. identified a specific type of periostin^+^ cancer-associated fibroblast that may promote lymph node metastasis in oral cancer by disrupting lymphatic endothelial barriers via the integrin-focal adhesion kinase/Src-VE-cadherin signaling pathway [151]. Li et al. elucidated that zinc finger protein 139 regulated annexin A proteins to accelerate lymph node metastasis in gastric cancer [152]. High expression of connexin-43 and E-cadherin was also found in the metastatic lymph nodes of gastric cancer [153]. Furthermore, an increase in enzymes such as lysyl oxidase, membrane type-matrix metalloproteinase and tissue inhibitor of matrix metalloproteinase 1 were associated with ECM degradation in the metastatic lymph nodes of patients with oral cancer [154]. In contrast, high levels of fibronectin, tenascin-C, and osteopontin in tumor stroma have been shown to be associated with lymph node metastasis [155, 156]. In summary, these studies indicate the importance of ECM remodeling in promoting lymph node metastasis.

### Therapeutic implication in targeting SLN

Because SLN involvement seems to be an initial and critical step for tumor metastasis, it could be a suitable target for therapeutic intervention by delivery of cytotoxic drugs or activation of immune response. Three strategies are discussed herein. First, reversal of metabolic alteration. As previously mentioned, metabolic changes in various cell types in the SLN are found to be important for the creation of a pre-metastatic microenvironment. Therefore, inhibition or restoration of the metabolic switch is a considerable approach. For example, stromal cells in the SLN preferentially utilize lipids as a major energy source and undergo FAO for ATP production. Suppressing FAO reaction locally in the SLN may reverse the microenvironment to normal immune active status to reduce cancer cell arrival and invasion. Second, anti-cancer drugs can be directly delivered to SLN or regional lymph nodes to kill cancer cells. The main challenge of this approach is the development of lymph node-tropic nanoparticle. The size and characteristic of nanoparticle are key determinants for specific lymph node targeting. It has been shown that nanoparticles ranging 5 to 50 nm in size are favorable for uptake by lymphatic capillary and vessel, thus promoting SLN entry [157, 158]. Magnetic drug-conjugated nanoparticle can be concentrated in regional lymph nodes using an external magnet [159, 160]. The design of a drug-delivery particle with high SLN tropism is an important issue in nanomedicine. Third, enhancement of anti-cancer immunity in the SLN by immune modulators and cancer vaccines. Immunotherapy has become one of the mainstreams in cancer treatment. However, only a small proportion of patients with cancer benefit from immune checkpoint inhibitors. Recent studies demonstrated that SLN and tumor-draining lymph nodes are potential targets for re-enforcing immune responses. Sasso et al. developed lymphangiogenic potentiation of immunotherapy by injecting of VEGF-C-overexpressing and adjuvant-covering killed cancer cells to elicit T cell activation at the injection sites and draining lymph nodes [161]. Francis et al. also showed that locoregional delivery of immune checkpoint inhibitors in lymph nodes triggered enhanced cancer immunotherapy in the animal model of melanoma [162]. Intranodal injection of tumor-specific antigens increases DC presentation and promotes the efficacy of cancer vaccines. These results suggest that targeting SLN or tumor-draining lymph nodes could be a new direction for the development of anti-cancer drugs.

## Conclusions

In a landing battle, the establishment of a beachhead is the most critical step in winning the war. To successfully disseminate to distant organs, cancer cells send many outpost troops to establish a beachhead in the SLN for further metastasis. These outpost troops could be macromolecules such as EVs, growth factors, and ECM proteins or small molecules such as miRNA and metabolites. These secreted factors remodel all cell types, including immune cells, LEC, BEC, and fibroblasts, in the SLN and change the foe to friend to create a favorable microenvironment for cancer cell invasion. Our understanding of SLN reprogramming has vastly improved in the past two decades. In the near future, we can expect the application of lymph node-targeted drugs and vaccines for cancer treatment.

## Data Availability

Not applicable.

## Abbreviations

BEC: Blood endothelial cell
CAF: Cancer associated fibroblast
CCL21: C–C motif Chemokine ligand 21
CCR: CC-chemokine receptor
COX: Cyclooxygenase
Cox7c: Cytochrome c oxidase subunit 7c
CPT1A: Carnitine palmitoyltransferase 1A
CTLA4: Cytotoxic T-lymphocyte-associated protein 4
CXCR: C-X-C motif chemokine receptor
2-HG: 2-hydroxyglutarate
DC: Dendritic cell
DEG: Differentially expressed gene
ECM: Extracellular matrix
ER: Estrogen receptor
EV: Extracellular vesicle
FAH: Fumarylacetoacete hydroxylase
FAK: Focal adhesion kinase
FAO: Fatty acid oxidation
FDC: Follicular dendritic cell
Foxp3: Forkhead box P3
FRC: Fibroblastic reticular cell
GM-CSF: Granulocyte macrophage-colony stimulating factor
HEV: High endothelial venules
IDO: Indoleamine 2,3-dioxygenase
IFN: Interferon
IL: Interleukin
LEC: Lymphatic endothelial cell
LOX: Lysyl oxidase
LTo: Lymphoid tissue organizer
LYVE-1: Lymphatic vessel endothelial hyaluronan receptor 1
M-CSF: Macrophage colony-stimulating factor
MDSC: Myeloid-derived suppressor cells
MEF2C: Myocyte-specific enhancer factor 2C
MHC: Major histocompatibility complex
Ndufv2: Ubiquinone oxidoreductase core subunit v2
OCR: Oxygen consumption rate
OXPHOS: Oxidative phosphorylation
PDGF: Platelet-derived growth factor
PDPN: Podoplanin
PGC-1α: Proliferator-activated receptor gamma coactivator 1 alpha
PGE2: Prostaglandin E2
PI3K: Phosphatidylinositol-3-kinase
PNAd: Peripheral node addressin
Prdx5: Peroxiredoxin-5
Prox-1: Prospero homeobox protein 1
SDHD: Succinate dehydrogenase complex Subunit D
SLN: Sentinel lymph nodes
STAT: Signal transducer and activator of transcription
TAM: Tumor-associate macrophage
TFAM: Transcription factor A, mitochondrial
TGF-β: Transforming Growth Factor Beta
Treg: Regulatory T cell
Uqcr11: Ubiquinol-cytochrome c reductase, complex III subunit XI
VEGF: Vascular endothelial growth factor
VEGFR-3: Vascular endothelial growth factor receptor 3
YAP: Yes-associated protein
